# Health systems supports for community case management of childhood illness: lessons from an assessment of early implementation in Malawi

**DOI:** 10.1186/1472-6963-13-55

**Published:** 2013-02-11

**Authors:** Jennifer A Callaghan-Koru, Kate Gilroy, Adnan A Hyder, Asha George, Humphreys Nsona, Angella Mtimuni, Bernie Zakeyo, Josiah Mayani, Cristina V Cardemil, Jennifer Bryce

**Affiliations:** 1 Department of International Health, Johns Hopkins Bloomberg School of Public Health, 615 N. Wolfe Street, Baltimore, MD, 21218, USA; 2 Department of Preventive Health, Malawi Ministry of Health, Lilongwe, Malawi; 3 Zomba, Malawi; 4 University of Malawi College of Medicine, Blantrye, Malawi; 5 Department of Pediatrics, Children’s National Medical Center, Washington, DC, USA

**Keywords:** Community case management, Community health workers, Child health, Malawi, Supervision, Drug supply, Job aids

## Abstract

**Background:**

National community-based health worker (CBHW) programs often face challenges in ensuring that these remote workers are adequately trained, equipped and supervised. As governments increasingly deploy CBHWs to improve access to primary health care, there is an urgent need to assess how well health systems are supporting CBHWs to provide high quality care.

**Methods:**

This paper presents the results of a mixed-methods assessment of selected health systems supports (supervision, drug supply, and job aids) for a national community case management (CCM) program for childhood illness in Malawi during the first year of implementation. We collected data on the types and levels of drug supply and supervision through a cross-sectional survey of a random sample of Health Surveillance Assistants (HSAs) providing CCM services in six districts. We then conducted in-depth interviews and focus group discussions with program managers and HSAs, respectively, to gain an understanding of the barriers and facilitating factors for delivering health systems supports for CCM.

**Results:**

Although the CCM training and job aid were well received by stakeholders, HSAs who participated in the first CCM training sessions often waited up to 4 months before receiving their initial supply of drugs and first supervision visits. One year after training began, 69% of HSAs had all essential CCM drugs in stock and only 38% of HSAs reported a CCM supervision visit in the 3 months prior to the survey. Results of the qualitative assessment indicated that drug supply was constrained by travel distance and stock outs at health facilities, and that the initial supervision system relied on clinicians who were able to spend only limited time away from clinical duties. Proactive district managers trained and enrolled HSAs’ routine supervisors to provide CCM supervision.

**Conclusions:**

Malawi’s CCM program is promising, but health systems supports must be improved to ensure consistent coverage and quality. Mixed-methods implementation research provided the Ministry of Health with actionable feedback that it is using to adapt program policies and improve performance.

## Background

The majority of low-income countries are not on track to meet Millennium Development Goal 4 of achieving a two-thirds reduction in child mortality by 2015, despite improved global efforts in maternal and child health [1]. Coverage of key child health interventions, such as artemisinin-combined therapies for malaria and antibiotics for pneumonia, remains low [2]. The WHO and UNICEF promote integrated community case management of childhood illnesses (CCM) as a strategy that countries can adopt to improve coverage levels of these key curative interventions [3]. CCM programs typically include the treatment of uncomplicated childhood illnesses in communities by trained volunteer or lay health workers, as well as timely referral for severe illnesses and counseling on health promotion and care seeking [4]. CCM has proven effective at reducing child mortality rates in controlled intervention trials and pilot programs [5-7], but there are fewer examples of integrated CCM programs at regional or national scale [8], especially in Africa [9]. Despite the growing interest in scaling-up CCM programs in many countries, implementing and sustaining support for these programs remains a major challenge in low-income settings and there has been little published research investigating implementation of routine CCM programs [10-12]. This has prompted calls for urgent research that explores how CCM programs can be implemented effectively [13-15].

CCM programs must be well supported by the health system in order to function effectively and achieve the desired impact on child health [4,16-18]. Following initial training in CCM, community-based health workers (CBHWs) should receive regular supportive supervision and consistent drug supplies, and be supported by effective job aids [10,17,18]. CBHWs providing CCM services often have no previous clinical experience, are trained for short periods of time, have low levels of education, and are posted in isolated settings [18-22]. As a result, supportive supervision, including observation of case management and corrective feedback, and effective job aids, are considered particularly important for ensuring high quality care by CBHWs [23,24]. Disruptions in supplies of essential drugs prevent CBHWs from providing life saving treatments, and can also damage the credibility of CCM programs and CBHWs among community members [10,25]. Lack of health system support contributed to the mixed results and decline of CBHW programs initiated following the Declaration of Alma Atta [19,20], thus it is crucial that health systems supports for CCM are addressed to ensure the success and sustainability of newly introduced CCM programs.

This paper examines the implementation of training, supervision, drug supply, and job aids during the scale-up of a national CCM program in Malawi. Malawi, a small low-income country in southeast Africa, was an early adopter and implementer of CCM [8]. Malawi’s under-five mortality rate is estimated at 92 per 1,000 live births [1]. Although Malawi is considered on-track to reach MDG 4, the country must further reduce the under-five mortality rate by 20% to reach its MDG target [1]. In 2008, as a part of its child health strategy, Malawi’s Ministry of Health (MOH) began training Health Surveillance Assistants (HSAs), an existing cadre of paid CBHWs, to treat uncomplicated cases of malaria, pneumonia, and diarrhea in the community [26,27]. The objectives of this paper are to: 1) describe the types and levels of selected health systems supports provided to HSAs performing CCM in Malawi; and, 2) identify factors that constrain and facilitate the delivery of health systems supports.

## Methods

Although the concept of health systems supports for CCM can be broadly conceptualized to include many factors, this study focuses on the primary concerns of the MOH in Malawi during early implementation of the CCM program: 1) implementation of training; 2) supervision; 3) drug supply; and 4) job aids. The focus of the study is based on the specific context of the CCM program in Malawi; other health systems factors, such as recruitment standards, compensation and incentives, training content, and engagement of the community, are tied to policies for the entire HSA cadre, rather than the introduction of CCM to HSAs’ activities, and are not addressed here.

### Study setting

Health Surveillance Assistants are a cadre of CBHW in Malawi that was originally established in the 1960s to support smallpox vaccinations [28]. HSAs are formal non-clinician health workers who are salaried by the government, are required to have 10 to 12 years of education, and undergo a 10-week basic training [29]. The government recruited over 5,000 new HSAs between 2002 and 2010 through grants from the Global Fund to Fight AIDS, Tuberculosis, and Malaria. HSAs now serve a catchment area of approximately 1,000 people and their main functions are to provide health education and sanitation within the communities where they are posted [29]. Although some HSAs serve urban areas, the majority are posted to rural communities and are supervised by Assistant Environmental Health officers (AEHOs)--non-clinicians under the MOH’s Preventive Health Section and based at health facilities–and Senior HSAs–HSAs posted in communities who have been promoted to a supervisory role. HSAs are expected to receive a supervision visit from an AEHO or Senior HSA in their community each month; additional supervision occurs during quarterly review meetings.

**Figure 1:**
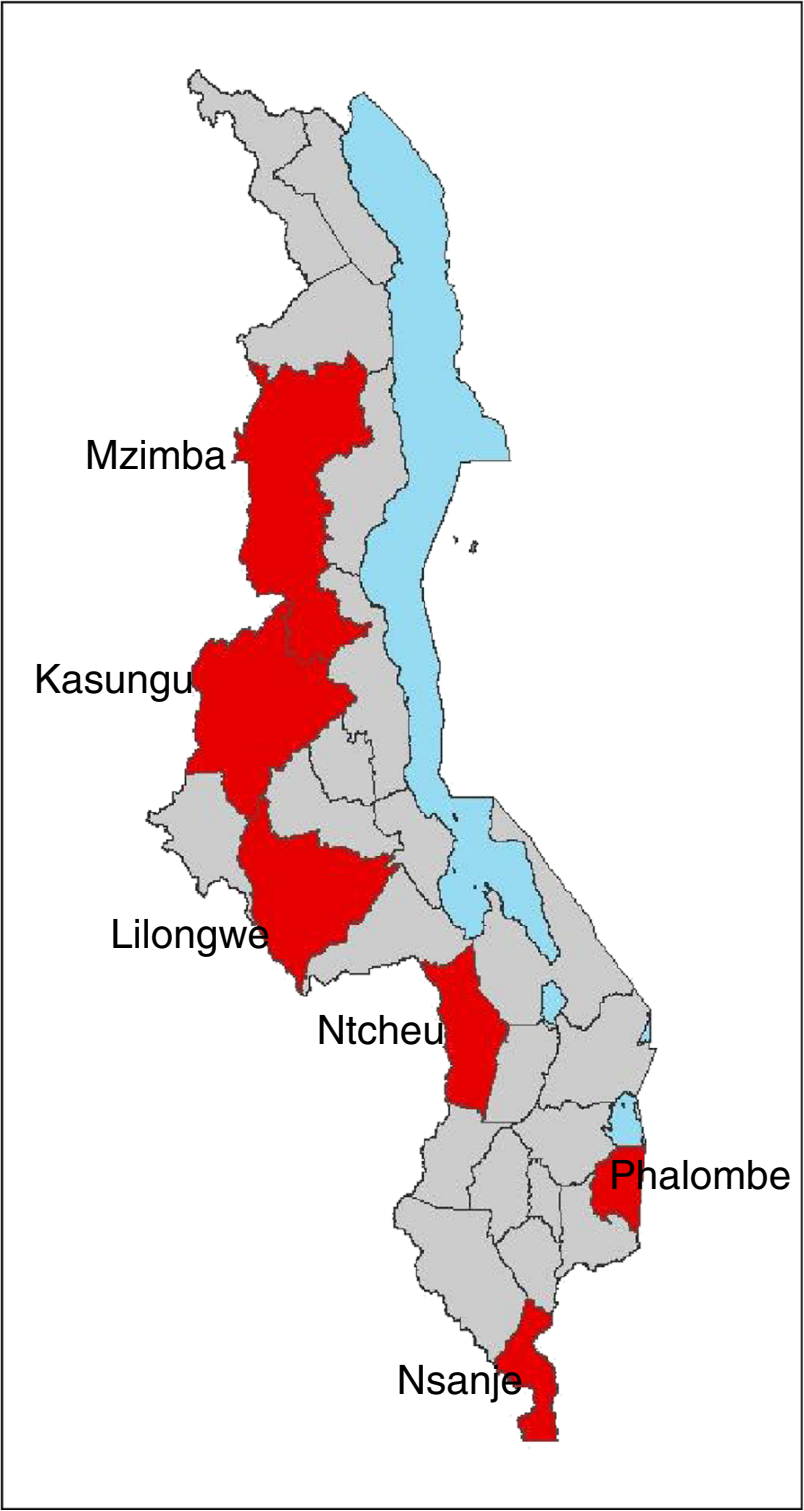
Map of study districts.

HSAs began to provide CCM services in Malawi in September 2008 in ten districts receiving technical support and funding from WHO and UNICEF for early CCM implementation. Selected HSAs received six days of training to treat uncomplicated cases of malaria, pneumonia, and diarrhea following a job aid, known as the sick child recording form, with an algorithm adapted from the Integrated Management of Childhood Illness (IMCI) guidelines [30]. The six-day trainings were led centrally at the district level by trained clinicians from district hospitals. Implementation plans specified that HSAs should receive a wooden drug box, with a lock and initial drug supplies, on the last day of training. Six of the ten early districts with the strongest levels of CCM implementation (based on the number of HSAs trained and equipped to operate village health clinics) were selected for inclusion in this early evaluation of the program. The six districts represent all three of Malawi’s regions–two from the southern region, three in the central region, and one in the northern region (see Figure 1). Facility-based health services in these districts are provided by the Malawi MOH, which provides services free of charge, and the Christian Health Association of Malawi (CHAM), a non-profit organization that charges user fees for most services and drugs provided at its clinics. Additional details about the HSA program and components of the CCM package are available elsewhere [29].

### Study design and data collection

This study was conducted in partnership with the Ministry of Health as a part of an independent evaluation of the Catalytic Initiative, led by the Johns Hopkins University Institute for International Programs and the Malawi National Statistics Office. The design followed an explanatory-sequential approach [31] by collecting quantitative data on the types and levels of health systems supports, followed by a qualitative study to gain greater understanding of the support systems, barriers, and facilitating factors. Quantitative data on the quality of care and the types and levels of health system supports were collected from a random sample of HSAs operating village health clinics in the six selected districts through a cross-sectional survey, with results on quality of care available elsewhere [32]. Data were collected by three-person survey teams, composed of CCM trainers from the Ministry of Health, trained and supervised by Johns Hopkins researchers and MOH managers. Briefly, the teams visited sampled HSAs in the rural posts where they conduct village health clinics to observe sick child consultations, record the availability of drugs, and to ask the HSA to recall the supervision visits he or she received and any drug stock outs in the past 3 months using a standard questionnaire. In order to minimize bias in data collection, survey teams were sent to different districts than those in which they worked. A sample size of 132 HSAs was calculated to allow observation of 369 sick child consultations and estimate the proportion of sick children correctly managed with +/− 6%, assuming 95% confidence and a design effect of 1.5 [32]. Data collection took place during a four-week period in October and November 2009, approximately one year after CCM implementation began. The indicators for drugs and equipment were selected based on the functional requirements of the village health clinic as indicated by the national CCM treatment algorithm [27] and were calculated for the overall sample and each district using Stata 10 [33]. Differences between districts were tested using Fischer’s exact test for proportions and the Kruskall-Wallis rank-sum test for interval indicators.

At the time of the study there had been no systematic documentation of how the CCM program was being implemented at district level to support interpretation of quantitative results. We therefore conducted follow up interviews and focus groups to obtain information on program implementation strategies and challenges, focusing on the areas of CCM training, supervision, job aids, and drug supply. These qualitative data were collected over a four-week period in November and December 2009 by two independent Malawian researchers and a Johns Hopkins researcher, with permission and introductions from Ministry of Health officials. A pilot of the qualitative protocol and interview guides, conducted in a district excluded from the study, identified the relevant personnel in each district to include the District Health Officer, IMCI Coordinator, Administrator, Environmental Health Officer, Assistant Environmental Health Officers, Health Center Clinicians, and Senior HSAs. Although the study team interviewed the IMCI Coordinators--the manager with primary responsibility for the CCM program at district level--for all six districts included in the survey, study timing did not permit visits to all districts. Four of the six districts--Lilongwe, Kasungu, Phalombe, and Ntcheu--representing high- and low-performing districts in terms of supervision and drug supply based on the preliminary results from the quantitative survey, were selected for in-depth interviews with other health personnel. In-depth investigation in these four districts was expected to reveal variations in implementation strategies and challenges between districts.

During visits to each of the four districts, the study team attempted to interview each of the individuals occupying these positions. Focus groups with HSAs operating village health clinics were also conducted in the four selected districts. Interview and focus group methods are described in detail elsewhere [34]. Three members of the research team coded all transcripts using a standard coding index. Analysis procedures for exploring themes in the data included summarizing health system strategies (e.g., drug supply delivery method) for each district, developing implementation timelines, and placing coded data in charts by district and respondent cadre. Reported constraints for delivering health system supports were analyzed for underlying factors using root cause analysis techniques [35]. Data from the quantitative and qualitative components of the study were triangulated, and associations between qualitative data on health systems strategies and quantitative outcomes were assessed for using Fischer’s exact test. Preliminary results were reviewed with stakeholders at national and district level, including representatives of DHMTs in all participating districts. This study was approved by the Institutional Review Boards at the Johns Hopkins Bloomberg School of Public Health and the Malawi National Health Sciences Research Committee.

## Results

A total of 131 HSAs were surveyed for drug supply, equipment, and supervision levels (Table 1). The majority of HSAs in the survey were male, had completed secondary school, and had spent less than five years in the community where they were working. Based on review of available registers, the median number of sick children treated by each HSA in September 2009 was 41. The qualitative sample included 4 focus group discussions with HSAs and interviews with 9 supervisors and clinicians and 20 district-level managers (Table 2). The results section is presented by thematic content (training and establishing supports, drug supply, supervision, and job aids) and draws on both the quantitative and qualitative results.

**Table 1:** Descriptive statistics for the sample of Health Surveillance Assistants

**District**	**All districts**	**Kasungu**	**Lilongwe**	**Mzimba**	**Nsanje**	**Ntcheu**	**Phalombe**
**Age**							
20 to 24	13 (9.9%)	2 (9.5%)	1 (4.6%)	1 (4.6%)	3 (13.6%)	4 (18.2%)	2 (9.1%)
25 to 29	37 (28.2%)	8 (38.1%)	3 (13.6%)	5 (22.7%)	5 (22.7%)	9 (40.9%)	7 (31.8%)
30 to 39	57 (43.5%)	10 (47.6%)	10 (45.5%)	7 (31.8%)	12 (54.6%)	8 (36.4%)	10 (45.5%)
40 to 49	19 (14.5%)	1 (4.8%)	5 (22.7%)	8 (36.4%)	1 (4.6%)	1 (4.6%)	3 (13.6%)
50 and above	5 (3.8%)	0	3 (13.6%)	1 (4.6%)	1 (4.6%)	0	0
**Sex**							
Male	106 (80.9%)	17 (80.9%)	18 (81.8%)	18 (81.8%)	16 (72.7%)	15 (68.2%)	22 (100.0%)
Female	25 (19.1%)	4 (19.1%)	4 (18.2%)	4 (18.2%)	6 (27.3%)	7 (31.8%)	0
**Recruitment year**							
Before 1990	2 (1.5%)	0	1 (4.6%)	1 (4.6%)	0	0	0
1990 to 1999	52 (39.7%)	8 (38.1%)	11 (50.0%)	13 (59.1%)	7 (31.8%)	5 (22.7%)	8 (36.4%)
2000 to 2004	13 (9.9%)	0	2 (9.1%)	1 (4.6%)	5 (22.7%)	0	5 (22.7%)
2005 to 2009	64 (48.9%)	13 (61.9%)	8 (36.4%)	7 (31.8%)	10 (45.5%)	17 (77.3%)	9 (40.9%)
**Education**							
Primary school	5 (3.8%)	0	2 (9.1%)	2 (9.1%)	0	0	1 (4.6%)
Form Two	45 (34.4%)	6 (28.6%)	11 (50.0%)	6 (27.3%)	13 (59.1%)	4 (18.2%)	5 (22.7%)
Form Four	81 (61.8%)	15 (71.4%)	9 (40.9%)	14 (63.6%)	9 (40.9%)	18 (81.8%)	16 (72.7%)
Years in community*							
Less than 1 year	19 (14.8%)	3 (14.3%)	3 (14.3%)	2 (9.1%)	7 (35.0%)	2 (9.1%)	2 (9.1%)
1 to 5 years	75 (58.6%)	14 (66.7%)	12 (57.2%)	15 (68.2%)	7 (35.0%)	14 (63.6%)	13 (59.1%)
5 to 10 years	22 (17.9%)	2 (9.5%)	4 (19.1%)	4 (18.2%)	2 (10.0%)	5 (22.7%)	5 (22.7%)
More than 10 years	8 (6.3%)	2 (9.5%)	2 (9.5%)	1 (4.6%)	0	1 (4.6%)	2 (9.1%)
From community	4 (3.1%)	0	0	0	4 (18.2%)	0	0
**Median number of CCM cases per HSA in September, 2009 (IQR)**							
	41 (54)	50 (65)	48 (45)	25 (52)	29 (54)	51 (48)	34 (64)

*n=128; 3 missing.

**Table 2:** Description of the interview and focus group data

**Cadre of worker**	**Type**	**No. participants**
Administrators	In-depth interviews	4
Assistant Environmental Health Officers	In-depth interviews	4
District Environmental Health Officers	In-depth interviews	2
District Health Officers	In-depth interviews	3
Health Surveillance Assistants	Focus group discussions (4 total)	29
Health Center In-Charges	In-depth interviews	3
IMCI Coordinators and Deputies	In-depth interviews	7
National-level stakeholders	In-depth interviews	6
Pharmacy Technicians	In-depth interviews	4
Senior HSAs	In-depth interviews	2
**TOTALS**		**64**

### Training and establishing supports for CCM

Following national workshops to adapt the CCM algorithm and train trainers in September 2008, the ten districts selected for early implementation started training with HSAs posted to the most “hard-to-reach” areas. Hard-to-reach areas are defined by each DHMT (usually the Environmental Health Officer), based on the general criterion offered by the MOH of approximately 7 km or farther from a health facility. DHMTs were responsible for determining the pace of training sessions and rollout of CCM in their district, with support and feedback from WHO and UNICEF offices. In qualitative assessments, managers from several districts indicated that they were purposefully introducing the program slowly in order to identify and correct problems with health system supports. As said by one district IMCI Coordinator: “It isn’t possible to just train everybody because we also have to consider the quality of the services they will be delivering . . . We suggest . . . to first sort out supervision and other logistics.”

**Figure 2:**
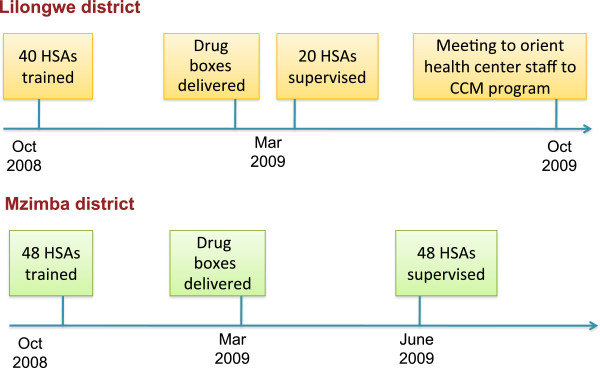
Timing of first trainings, drug distribution, and supervision in two districts.

Despite awareness of the need for supervision and other health system supports among some managers, these inputs and processes were still under development throughout the first year of the CCM program. Following the 6-day CCM training, HSAs were to begin operating their village health clinics (VHCs) when they received their wooden drug box and initial supply of drugs. The initial drug boxes were delivered directly to the HSAs’ post or the nearest health center from the district level. In most districts, there were substantial delays between the time that the HSAs completed training and when they received their drug box (Figure 2). Four districts (Lilongwe, Kasungu, Phalombe, and Mzimba) each had 40 or more HSAs trained by November 2008 who did not receive drug boxes until March 2009. After the delivery of the initial drug supply with the drug box, the MOH’s strategy was to integrate CCM drug resupplies into the routine drug supply system. HSAs disbursed the same drugs, in the same packaging, as health facilities, and were to collect resupplies of drugs from their nearest health facility, which in turn requisitioned resupplies from national and regional medical stores.

During the CCM program’s first year, the national IMCI office had not yet provided guidance on supervision protocols to districts, such as standardized checklists or guidelines for activities to be conducted during supervision. District IMCI coordinators reported a common understanding in the qualitative interviews that the expectations from the national level were that HSAs should receive a follow-up visit in their communities within 6 weeks of training, followed by monthly CCM-specific supervision visits. However, similar to provision of drug supplies, implementation of supervision visits was delayed in most districts. None of the six districts began CCM supervision visits earlier than 4 months after the first HSAs were trained, and some HSAs did not receive any supervision visit for 8 months following training.

### Drug supply at one year

Three of the six drugs included in the CCM algorithm in Malawi--cotrimoxazole, Coartem (Lumefantrine-artemether, an antimalarial combination therapy),^a^ and oral rehydration solution (ORS)--are critical for treatment of the priority childhood illnesses of malaria, pneumonia, and diarrhea, and are therefore the focus of this analysis^b^. Among the 131 HSAs enrolled in the survey, 68.7% [CI: 60.0, 76.5] had all three critical drugs available at their respective CCM clinics at the time of the survey team’s visit (Table 3). The percentage of HSAs with all three drugs on the day of the survey team visit ranged from 100% in Nsanje [CI: 84.5, 100.0] to 52.4% in Kasungu [CI: 29.8, 74.3]. When asked to recall stock outs in the previous 3 months, 46.6% of HSAs reported having a stock out of any one of the three critical drugs [CI: 37.8, 55.5]. HSAs reported that Coartem, the only drug which is provided to HSAs in age-specific dosing, was frequently out of stock in the dosage for children aged 36 to 60 months (2x6), with 45.8% [CI: 37.1, 54.7] reporting a stock out in the past 3 months. Over 40% of HSAs also reported a stock out of ORS [42.7%; CI: 34.1, 51.7] but only 10.7% [CI: 6.0, 17.3] of HSAs reported a Cotrimoxazole stock out in 3 months (Additional file 1: Table S2).

**Table 3:** Drug supply, stock outs, and resupply mechanisms

**Indicator**	**n**	**All Districts**	**Kasungu**	**Lilongwe**	**Mzimba**	**Nsanje**	**Ntcheu**	**Phalombe**	**p-value**
Proportion of HSAs with all critical drugs for CCM—ACTs, cotrim, and ORS—on the day of the visit (95% CI)	131	68.7 (60.0, 76.5)	52.4 (29.8, 74.3)	63.6 (40.7, 82.8)	45.5 (24.4, 67.8)	100 (84.5, 100.0)*	90.9 (70.8, 98.9)	59.1 (35.4, 79.3)	0.000
Proportion of HSAs with cotrimoxazole on the day of the visit (95% CI)	131	96.2 (91.3, 98.7)	85.7 (63.7, 97.0)	95.5 (77.2, 99.9)	85.7 (63.7, 97.0)	100 (84.6, 100.0)*	100 (84.6, 100.0)*	100 (84.6, 100.0)*	0.111
Proportion of HSAs with ACTs (Coartem, any dose) on the day of the visit** (95% CI)	131	93.1 (87.4, 96.8)	81.0 (58.1, 94.6)	95.5 (77.2, 99.9)	86.4 (65.1, 97.1)	100 (84.6, 100.0)*	100 (84.6, 100.0)*	95.6 (77.2, 99.9)	0.074
Proportion of HSAs with ORS on the day of the visit (95% CI)	131	74.0 (65.7, 81.3)	71.4 (47.8, 88.7)	68.2 (45.1, 86.1)	54.5 (32.2, 75.6)	100 (84.6, 100.0)*	90.9 (70.8, 98.9)	59.1 (36.4, 79.3)	0.003
Proportion of HSAs who report a stockout of any critical drugs in the last 3 months*** (95% CI)	131	46.6 (37.8, 55.5)	66.7 (43.0, 85.4)	50.0 (28.2, 71.8)	50.0 (28.2, 71.8)	0 (0, 15.4)*	40.9 (20.7, 63.6)	72.7 (49.8, 89.2)	0.000
Median days duration of drug stock outs, any drug (IQR)	131	30 (49.3)	30 (46)	41 (75.8)	60 (82.5)	__	30 (15)	30 (46)	__
Proportion of HSAs who received a resupply of drugs in the last 3 months (95% CI)	130	85.4 (78.1, 91.0)	95.2 (76.2, 99.9)	85.7 (63.7, 97.0)	68.2 (45.1, 86.1)	86.4 (65.1, 97.1)	95.5 (76.2, 99.9)	81.8 (959.7, 94.8)	0.114
Among HSAs who restocked in the last 3 months, proportion who restocked at the nearest health center (95% CI)	111	81.1 (72.5, 87.9)	95.0 (75.1, 99.9)	100.0 (81.5, 100)*	93.9 (68.1, 99.8)	84.2 (60.4, 96.6)	28.6 (11.3, 52.2)	94.4 (72.7, 99.9)	0.000

P-values for differences in proportions between districts are calculated using a chi-squared test; P-values for stock out days are calculated using Kruskall-Wallis rank-sum test; *One-sided 97.5% confidence interval; **Stock out of all Coartem considered when both versions of Coartem stocked out for 45 days or more. ***Stock outs in the last 90 days include observations where a drug was never received.

Among the 111 HSAs who received a resupply of drugs in the prior 3 months, the majority restocked at the health center [81%; CI: 72.5, 87.9]. The location of drug resupply in the three months previous to the survey also varies significantly by district. Lilongwe is the only district where 100% of resupplied HSAs received their resupply from a health center, while 81% of HSAs restocked at the district hospital in Ntcheu district and 39% of HSAs received drug stocks from a visiting supervisor in Phalombe (Additional file 1: Table S2).

Informants reported important challenges in implementing the official drug supply strategy for the CCM program, which stipulates that HSAs should visit their nearest health center to request drug stocks (Table 4). Health center clinicians in several districts reported that they resisted supplying HSAs with drugs at first because they had not been informed about the CCM program and drug supply policies. Additionally, informants at all levels noted that the health centers themselves experience periodic drug shortages and stock outs. When drug stocks at the health center are inadequate, clinicians are unable or unwilling to provide scarce supplies to HSAs. According to a health center in-charge, “The only problem that there is about drug supply [is] we don’t have drugs here at the health center.” HSAs in some districts indicated that mistrust had developed between themselves and health center clinicians over the sharing of drug supplies. One HSA stated: “In my opinion I feel that it is just the selfishness of those medical assistants. They say ‘we have no drugs’ but they are operating their services so it is very difficult to say that this problem is from central medical stores to health facilities.”

**Table 4:** Challenges and solutions for drug supply and supervision identified by managers and HSAs

**Challenges identified by participants**	**Solutions implemented by districts**
**Drug supply**	
• Distance from HSA posts to health facilities and lack of transport	• Pharmacy technicians deliver drugs to HSAs at their posts
	• District managers allow HSAs to collect emergency supplies of drugs from the district hospital stores
• HSAs working in the catchment areas of health facilities operated by the Christian Health Association of Malawi (CHAM) are unable to collect drugs from their nearest facility due to conflicts in user fee policies	• District managers reach an agreement to reimburse CHAM for drugs supplied to HSAs
• Resistance by health center clinicians to supplying drugs to HSAs when they are unaware of the new program	• Orientation sessions held for health center clinicians
**Supervision**	
• District-level managers lack time to make supervision visits to HSAs’ communities	• Assistant environmental health officers and Senior HSAs are included in CCM trainings to enable them to conduct CCM supervision
• Managers have difficulty securing vehicles and fuel for supervision visits	
• Managers lack clear guidelines on what activities should be conducted during supervision visits	• District managers develop their own supervision checklist, with assistance of partner organizations

Distance was a barrier to drug supply for many HSAs who reported difficulties traveling to the health center to collect drugs and in some cases spending their own money for transport. Transport difficulties exacerbate the challenge with drug stocks at health centers, as expressed by one HSA: “When we go to the government facility, most of the times drugs are out of stock and this is an embarrassment to us because we travel a long distance.” The challenge of distance was even greater for HSAs reporting to health centers managed by CHAM, who were expected to travel to the nearest government health center to collect drugs which often was further away than the CHAM facility serving their catchment area.

In several districts, managers took various initiatives to try to reduce the constraints to drug supply. In some cases, these practices served to bolster the official drug supply policy, and in other cases the practices were outside the official policy. In support of the official policy, districts undertook activities that increased the involvement in or awareness of the CCM program among health center staff. Three districts held one-off review or orientation meetings to inform the health center clinicians about the CCM program, while another district included the clinicians during the initial community sensitization activities conducted by the district-level managers. Practices that also facilitated drug supply, but were outside of the official drug supply policy, included: 1) allowing HSAs to collect drugs directly from the district hospital; 2) delivering drugs to the HSAs in their communities; and 3) establishing agreements with facilities operated by CHAM. These efforts, although positive, did not show a significant association with drug supply or drug stock-outs in this study.

### Supervision at one year

Table 5 presents results related to the frequency and type of supervision received by the sampled HSAs. Overall, levels of routine CCM supervision reported by HSAs were low. Within the period of 3 months prior to the survey, less than 60% of the HSAs reported receiving any type of supervision visit [58.6%, CI: 49.6, 67.2], and only about 40% reported a visit specific to CCM [38.3%; CI 29.8, 47.3]. Moreover, only 15.6% of HSAs received a CCM supervision visit that included the supervisor observing the HSA while managing a sick child.

**Table 5:** Frequency and type of supervision received by surveyed HSAs

**Indicator**	n*	**All Districts**	**Kasungu**	**Lilongwe**	**Mzimba**	**Nsanje**	**Ntcheu**	**Phalombe**	**p-value**
Proportion of HSAs who received ANY supervision visit in the last month (95% CI)	127	27.6 (20.0, 36.2)	47.6 (25.7, 70.2)	19.0 (5.4, 41.9)	54.5 (32.2, 75.6)	22.7 (7.8, 45.4)	9.1 (1.1, 29.2)	10.5 (1.3, 33.1)	0.001
Proportion of HSAs who received ANY supervision visit in the last 3 months (95% CI)	128	58.6 (49.6, 67.2)	61.9 (38.4, 81.9)	45.0 (23.1, 68.5)	68.2 (45.1, 86.1)	45.5 (24.4, 67.8)	63.6 (40.7, 82.8)	66.7 (43.0, 85.4)	0.439
Proportion of HSAs who received a follow-up supervision within 6 weeks of CCM training (95% CI)	128	23.4 (16.4, 31.7)	10.0 (1.2, 31.7)	28.6 (1.3, 52.2)	38.1 (18.1, 61.6)	9.1 (1.1, 29.2)	31.8 (13.9, 54.9)	22.7 (7.8, 45,4)	0.136
Proportion of HSAs who received a supervision visit specific to CCM in the previous 1 month (95% CI)	127	15.3 (9.6, 22.6)	14.3 (3.0, 36.3)	5.0 (0.1, 24.9)	36.4 (17.2, 59.3)	13.6 (2.9, 34.9)	22.7 (7.8, 45.4)	0 (0, 16.1)**	0.018
Proportion of HSAs who received a supervision visit specific to CCM in the previous 3 months (95% CI)	128	38.3 (29.8, 47.3)	23.8 (8.2, 47.2)	10.0 (1.2, 31.7)	59.1 (36.4, 79.3)	27.2 (10.7, 50.2)	40.9 (20.7, 63.6)	66.7 (43.0, 85.4)	0.001
Proportion of HSAs who received a CCM supervision in the previous 3 months that included observation of case management (95% CI)	128	15.6 (9.8, 23.1)	0 (0, 16.1)**	4.5 (0.1, 22.8)	22.7 (7.8, 45.4)	13.6 (2.9, 34.9)	9.1 (1.1, 29.2)	40.9 (20.7, 63.6)	0.002
Proportion of HSAs who discussed their CCM work with a supervisor at the health facility (95% CI)	129	44.2 (35.5, 53.2)	61.9 (38.4, 81.9)	23.8 (8.2, 47.2)	50.0 (28.2, 71.8)	18.2 (5.1, 40.3)	42.9 (21.8, 66.0)	68.2 (45.1, 86.1)	0.003

*Among the 131 HSAs included in the sample, observations for these indicators are excluded in the cases where the HSA reports not being in the community during part or all of the study period, responds “don’t know,” or data was missing from the survey form.

**One-sided 97.5% confidence interval.

Among the 49 HSAs reporting supervision specific to CCM in the previous 3 months, IMCI coordinators were the most frequently-cited cadre conducting the supervision visit. Some HSAs also reported that Assistant Environmental Health Officers, IMCI trainers, and pharmacy technicians provided CCM supervision (data not shown). The most commonly reported supervisors for the 75 HSAs receiving any supervision visit in the previous 3 months were Environmental Health Officers (30%) and Senior HSAs (21%). HSAs who received a CCM supervision visit in their communities reported that, during the supervisory visit, supervisors checked records (83.7% percent of visits), corrected the HSA’s work (71.4%), answered the HSA’s questions (67.3%), and provided positive feedback (63.3% of visits). Observation of the HSA performing case management was the least frequently conducted activity by supervisors (36.7% of supervision visits).

Although the MOH initially advocated monthly supervision of each VHC, the survey results demonstrated that supervision visits were in fact far less frequent. Every IMCI Coordinator interviewed for this study acknowledged that their district was unable to meet this standard of supervision, and the majority considered monthly supervision to be an unattainable target. Common supervision challenges cited by manager informants included supervisors’ busy schedules, lack of transportation, and the coordination required to undertake supervision in teams (teams often included Pharmacy Technicians, Environmental Health Officers, and Malaria Coordinators). Districts teams typically visited as many HSAs as possible during a one- to two-week period set aside for supervision and rarely spent enough time at each site to observe the HSA managing a sick child. In order to improve supervision frequency, two districts provided CCM training to routine HSA supervisors, such as Assistant Environmental Health Officers and Senior HSAs. However, supervision frequency was not significantly higher in these districts.

Both HSAs and managers agreed that supervision is valuable for the program, and that the current levels of supervision are inadequate. However, HSAs desired different outcomes from supervision than those expected by IMCI coordinators, Environmental Health Officers, and other managers. Program managers generally considered supervision as an opportunity to correct any mistakes that HSAs may be making: “for them to do a quality job throughout, they need to be supervised frequently . . . because if we leave them without supervision, they may miss some concepts.” The majority of HSAs welcomed opportunities for receiving feedback to “know whether [we] are doing fine or not”, but also expressed a strong desire for supervisors to help them solve the problems encountered while operating CCM clinics, particularly in terms of supplies and resource needs. The lack of problem solving during supervision visits resulted in frustration among HSAs, as expressed by one focus group participant:

“When they came to supervise me I told them what I was lacking in my clinic, but until now there is nothing that has changed. So I feel like I have not been supervised because when you are supervised you expect to see some change.” (HSA, Focus Group #4)

### Job Aid: the Sick Child Recording Form

A key finding that emerged from the qualitative data on health systems supports for CCM in Malawi is the importance of the job aid developed for the program, known as the Sick Child Recording Form (SCRF). This form was adapted in Malawi based on a similar form developed by WHO as a standard part of IMCI training and implementation [36]. During the CCM training, HSAs are taught to conduct assessment following steps on the form, and to make treatment decisions using the decision rules included on the form. Informants in the qualitative study had overwhelmingly positive opinions about the SCRF. Additionally, in over 90% of sick child consultations observed during the quality of care survey study, HSAs made reference to a hard copy of the SCRF while managing sick children. HSAs reported that they liked using the SCRF for their CCM work, as the following comments by HSAs illustrate:

“I like using the guide line chart because it acts as my sign post. Whenever I am confused, I consult it to know where I am lost and then I am in a better position to do what I am supposed to.” 

“I should say that the Sick Child Recording Form is the mwini filimu (the major actor) in this program.”

Managers also considered the SCRF to be important for the quality of CCM services. One IMCI coordinator said, “I think HSAs are doing a good job, and basically it is because they are using the Sick Child Recording Form.”

## Discussion

These results highlight the achievements and challenges faced by the Malawi MOH and partners as they worked to rapidly scale up a CCM program to expand access to curative services for under-fives in a large rural population. In addition to the six districts addressed in this case study, training and implementation was taking place simultaneously in the majority of Malawi’s districts. The MOH reported that by 2009, over 1,000 HSAs had received the six-day CCM training. The SCRF, which serves as the basis of the training and the job aid for CCM, was well received by all stakeholders and can be considered a critical element in the program’s success. The high levels of support for the program provided by partner organizations, in terms of technical assistance and funds for training and supervision, facilitated the rapid scale up.

These findings also highlight several areas for program improvement. Early assessments of CBHW programs from the 1980s indicated the need to plan for and deliver necessary health system supports for CBHW programs [17,19]. However, this evaluation illustrates how large-scale CCM programs, while expanding access, can be stymied by the lack of effective health system support. Inadequacies in the initial implementation plans and policies presented important barriers to the scale-up of CCM. Although drug supply strategies integrated well with existing systems, drug availability remained a challenge. Additionally, the initial supervision strategy was not clearly defined or sustainable and district-level program managers faced significant barriers in following the supervision expectations.

This study was nested in an early study of the quality of care provided by HSAs, so districts included in this study were chosen based on adequate implementation strength [37]. Other districts are likely to have weaker implementation of health system supports during the same calendar period. That said, the levels of health systems supports documented in this case study raise concerns about whether Malawi’s CCM program will achieve the expected increases in coverage levels for treatment of pneumonia, malaria, and diarrhea.

The health systems support with the most direct impact on effectiveness and coverage of the program is drug supply. We found that one-third of HSAs did not have all of the essential CCM drugs in stock on the day of the visit, and analysis of clinical errors revealed that over half of mismanaged fever and diarrhea cases were due to the HSA not having stock of the required treatments [32]. The most important barrier identified by informants--general drug availability at the restocking points for CHWs--is statistically correlated with drug availability among HSAs in Malawi [38]. Health center drug supply is a large health systems problem in Malawi, although accurate data measuring the frequency and severity of drug stock outs is limited. The government cites “lengthy procurement processes, poor specifications, weak logistical information systems, inadequate and unpredictable funding for medicines and inadequate infrastructure,” districts overspending on private sector drug purchases, and shortage of pharmaceutical staff as causes of drug stock outs [39]. Solving larger drug supply problems will take coordinated efforts at institutional reform and systems strengthening by many stakeholders [40]. At the same time, CCM program managers can make targeted improvements to factors under their control, such as providing supply chain training to CHWs and managers and addressing transportation barriers [39].

Just as districts struggled to provide consistent drug supplies, they were similarly unable to provide regular supervision with coaching on clinical skills. The focus in Malawi on the administrative side of supervision (e.g., checking records) is consistent with practices reported in other low-income countries [41]. However, these results raise concerns about whether the inability of supervisors to address problems and complaints might reduce HSAs’ motivation, as supportive supervision is important for morale [42-44]. In fact, HSAs expressed a desire for more assistance and problem solving from supervisors and for an expansion of their clinical role, while program managers in Malawi viewed CCM as a limited mandate for HSAs [34]. Sustaining supervision is a common challenge for both facility and community-based programs in weak health systems [41,45]. The initial supervision strategy in the Malawi CCM program of adding CCM supervision to the workload of busy facility-based clinicians, which is common in other programs [43,46,47], may be an unrealistic approach for regular and sustained supervision in many settings. Although CCM programs are often implemented in environments with constrained resources, planning for resources to implement effective supervision strategies is essential for scale-up [24]. Supervision initiatives that include training for supervisors in quality assurance, problem solving and coaching may improve performance and motivation [48-51].

Finally, an important finding of this study is the initiative and capacity demonstrated by some district health managers to adapt implementation strategies in order to overcome challenges. Health systems in LICs face considerable challenges in supporting CCM and other community-based programs. It is therefore encouraging to see that dedicated program managers can develop and gain support for strategies to address some of the barriers that they face, such as overcoming CCM supervision barriers through the involvement of HSAs’ routine supervisors. Although none of the reported strategies were statistically associated with better drug supply or supervision, this lack of association is possibly due to the relatively recent implementation of these strategies at the time of data collection. In the future, program implementers may consider fostering this type of decentralized problem solving through adaptive and flexible scale-up strategies [52]. Such efforts could potentially lead to solutions to local problems, as well as generalizable strategies that can be learned from and adopted across a program [52,53].

## Conclusions

There is growing concern among public health practitioners that weaknesses in implementation may result in failure to meet health targets such as the Millennium Development Goals [54]. Program coordinators in Malawi indicated that they have already begun to use the results of this study to target improvements in health systems supports in order to maximize the impact of CCM towards achieving their child health goals. In the broader policy context, this research reinforces the importance of moving beyond a train-and-deploy strategy towards a broader program development approach that includes training as one part of several strategic health systems supports required for successful scale-up. In terms of future research, CCM programming could benefit greatly from a broad research agenda including exploratory work to further elaborate bottlenecks to supports and quality of CCM as well as controlled trials testing different quality assurance strategies. A strong CCM research agenda is critical for ensuring that CBHW programs meet their potential and are sustained into the future.

## Endnotes

^a^ Coartem is supplied in blister packs with separate blisters for doses appropriate for ages 2 to 11 months (6 doses of 1 tablet, or 1x6) and ages 12 to 48 months (6 doses of 2 tablets, or 2x6). For the combined indicator of presence of all critical drugs, HSAs are considered to have Coartem if they have either the 1x6 or 2x6 blister packs as in practice these blister packs can be adjusted for either age group (e.g., providing two 1x6 blister packs to a child over 12 months).

^b^ Paracetamol, antibiotic eye ointment, and zinc are included in the CCM algorithm but not reported here. Zinc was not routinely available at the time of our study and therefore not included in our analysis. Paracetamol and eye ointment are not included in the priority indicators for treatment of malaria, pneumonia, and/or diarrhea. The three drugs excluded from this analysis are: paracetamol, antibiotic eye ointment, and zinc.

## Abbreviations

CCM: Community Case Management; CBHWs: Community-based Health Workers; CHAM: Christian Health Association of Malawi; DHMT: District Health Management Team; HSA: Health Surveillance Assistant; IMCI: Integrated Management of Childhood Illness; MOH: Ministry of Health; SCRF: Sick Child Recording Form; WHO: World Health Organization.

## Competing interests

Humphreys Nsona and Angella Mtimuni are members of the Ministry of Health office responsible for implementing iCCM in Malawi, but we have no other conflicts of interest to report.

## Authors’ contributions

JC, KG, and JB conceived of the study. CC, KG, and JC designed the quantitative study and instruments with input from JB, HN, and AM. JC designed the qualitative study with input from AG, KG, AH, JB, HN, and AM. JC oversaw data collection, lead data analysis, and wrote the first draft. JM and BZ collected qualitative data and participated in analysis and interpretation. HN and AM helped to supervise data collection and provided analysis feedback. AH, KG, JB and AG commented on early drafts. All authors contributed to interpretation of results and commented on drafts prior to publication. All authors read and approved the final manuscript.

## Pre-publication history

The pre-publication history for this paper can be accessed here:

http://www.biomedcentral.com/1472-6963/13/55/prepub

## Supplementary Material

Additional file 1: Table S1 Demographic and health profile of included districts. **Table S2.** Additional drug supply indicators. Click here for file
