# Supplementary material for: Health systems supports for community case management of childhood illness: lessons from an assessment of early implementation in Malawi
Source: BMC Health Serv Res. 2013 Feb 11;13:55. doi: 10.1186/1472-6963-13-55 (PMC3637472; doi:10.1186/1472-6963-13-55)
Supplement: Additional file 1: Table S1 — Demographic and health profile of included districts. Table S2. Additional drug supply indicators. [file 1472-6963-13-55-S1.docx]

**Additional Table 1: Demographic and health profile of included districts**

| **District** | **National** | **Kasungu** | **Lilongwe** | **Mzimba** | **Nsanje** | **Ntcheu** | **Phalombe** |
| --- | --- | --- | --- | --- | --- | --- | --- |
| Population [b] | 13,066,320 | 616,085 | 1,228,146 | 724,873 | 238,089 | 474,464 | 313,227 |
| Region | __ | Central | Central | Northern | Southern | Central | Southern |
| Land area (km^2^) [b] | 94,276 | 7,878 | 5,703 | 10,382 | 1,942 | 3,424 | 1,394 |
| Population density [b] | 139 | 78 | 215 | 70 | 123 | 139 | 225 |
| District hospital [c] | __ | Yes | Yes | Yes | Yes | Yes | No |
| Number of health facilities [c] | __ | 25 | 60 | 54 | 14 | 39 | 14 |
| Under-five mortality rate (deaths per 1,000) | 122 | 132 | 166 | 115 | 171 | 149 | 161 |
| Total fertility rate | 6.3 | 6.1 | 6.8 | 5.3 | 6.7 | 5.6 | 6.9 |
| Under-five stunting (-2 SD) | 0.46 | 0.469 | 0.461 | 0.462 | 0.385 | 0.504 | 0.468 |
| Under-five wasting (-2 SD) | 0.035 | 0.019 | 0.05 | 0.031 | 0.041 | 0.034 | 0.041 |
| Source: Multiple Indicator Cluster Survey 2008; [b] Malawi 2008 Census; [c] Malawi Ministry of Health. | | | | | | | |

**Additional Table 2: Additional drug supply indicators**

| **Indicator** | **Overall  (unadjusted)** | **Kasungu** | **Lilongwe** | **Mzimba** | **Nsanje** | **Ntcheu** | **Phalombe** | **p-value** |
| --- | --- | --- | --- | --- | --- | --- | --- | --- |
| **Drug stock out indicators** | | | | | | | | |
| Proportion of HSAs who report a stockout of cotrimoxazole in the last 3 months | 10.7 (6.0, 17.3) | 28.6 (11.3, 52.2) | 13.6 (2.9, 34.9) | 18.2 (5.2, 40.3) | 0 (0, 15.4)* | 4.5 (0.1, 22.8) | 0 (0, 15.4)* | 0.012 |
| Proportion of HSAs who report a stockout of ACTs (Coartem, both doses) in the last 3 months | 33.6 (25.6, 42.4) | 42.9 (21.8, 66.0) | 31.8 (13.9, 54.9) | 31.8 (13.9, 54.9) | 40.9 (20.7, 63.6) | 31.8 (13.9, 54.9) | 22.7 (7.8, 45.4) | 0.762 |
| Proportion of HSAs who report a stockout of ORS in the last 3 months | 42.7 (34.1, 51.7) | 52.4 (29.8, 74.3) | 40.9 (20.7, 63.6) | 50.0 (28.2, 71.8) | 0 (0, 15.4)* | 36.4 (17.2, 59.3) | 77.3 (54.6, 92.2) | 0.000 |
| **Drug resupply indicators** | | | | | | | | |
| Among HSAs who restocked in the last 3 months, proportion who restocked at the district hospital (n=111) | 21.6 (14.4, 30.4) | 5.0 (0.1, 24.9) | 0 (0, 18.5)* | 6.7 (0.2, 31.9) | 21.1 (6.1, 45.6) | 81.0 (58.1, 94.6) | 5.6 (0.1, 27.3) | 0.000 |
| Among HSAs who restocked in the last 3 months, proportion who restocked from visiting supervisor (n=111) | 9.0 (4.4, 15.9) | 0 (0, 16.8)* | 0 (0, 18.5)* | 0 (0, 21.8)* | 0 (0, 17.6)* | 14.3 (3.0, 36.3) | 38.9 (17.3, 64.3) | 0.000 |
| Among HSAs who restocked in the last 3 months, proportion who restocked from district hospital OR visiting supervisor (n=111) | 27.9 (19.8, 37.2) | 5.0 (0.1, 24.9) | 0 (0, 18.5)* | 6.7 (0.2, 31.9) | 21.1 (6.1, 45.6) | 85.7 (63.7, 97.0) | 38.9 (17.3, 64.3) | 0.000 |
| P-values for differences in proportions between districts are calculated using a chi-squared test; P-values for stock out days are calculated using Kruskall-Wallis rank-sum test; *One-sided 97.5 % confidence interval; **Stock outs in the last 30 days include observations where a drug was never received. Stock out of all LA considered when both versions of LA stocked out for 45 days or more. | | | | | | | | |
